# Depression among Asian-American Adults in the Community: Systematic Review and Meta-Analysis

**DOI:** 10.1371/journal.pone.0127760

**Published:** 2015-06-01

**Authors:** Hee Jun Kim, EunMi Park, Carla L. Storr, Katherine Tran, Hee-Soon Juon

**Affiliations:** 1 School of Nursing, University of Maryland, Baltimore, Maryland, United States of America; 2 School of Medicine, Johns Hopkins University, Baltimore, Maryland, United States of America; 3 Department of Family and Community Health, University of Maryland School of Nursing, Baltimore, Maryland, United States of America; 4 Johns Hopkins University, Baltimore, Maryland, United States of America; 5 Division of Population Science, Department of Medical Oncology, Thomas Jefferson University, Philadelphia, PA, United States of America; University of Vienna, AUSTRIA

## Abstract

**Objectives:**

In this systematic review, we provide an overview of the literature on depression among Asian-Americans and explore the possible variations in depression prevalence estimates by methodological and demographic factors.

**Methods:**

Six databases were used to identify studies reporting a prevalence estimate for depression in Asian-American adults in non-clinical settings. Meta-analysis was used to calculate pooled estimates of rates of depression by assessment type. Statistical heterogeneity was assessed for subgroup analyses by gender, age, ethnicity, and other participant characteristics.

**Results:**

A total of 58 studies met the review criteria (n = 21.731 Asian-American adults). Heterogeneity across the studies was considerably high. The prevalence of major depression assessed via standardized clinical interviews ranged between 4.5% and 11.3%. Meta-analyses revealed comparable estimated prevalence rates of depression as measured by the Center for Epidemiologic Studies Depression Scale (35.6%, 95% CI 27.6%–43.7%) and the Geriatric Depression Scale (33.1%, 95% CI 14.9%–51.3%). Estimates varied by Asian racial/ethnic group and other participant characteristics. Estimates of depression among special populations, which included maternity, caregivers, and homosexuals, were significantly higher than estimates obtained from other samples (58.8% vs 29.3%, p = .003). Estimates of depression among Korean and Filipino-Americans were similar (33.3%-34.4%); however, the estimates were twice as high as those for Chinese-Americans (15.7%; p = .012 for Korean, p = .049 for Filipino).

**Conclusion:**

There appears to be wide variability in the prevalence rates of depression among Asian-Americans in the US. Practitioners and researchers who serve Asian-American adults need to be sensitive to the potential diversity of the expression of depression and treatment-seeking across Asian-American subgroups. Public health policies to increase Asian-American access to mental health care, including increased screening, are necessary. Further work is needed to determine whether strategies to reduce depression among specific Asian racial/ethnic groups is warranted.

## Introduction

Depression is one of the most prevalent health issues worldwide. While Major Depressive Disorder (MDD) places a considerable burden on both society and the individual, poor health and impaired functioning are also found to be associated with depressive symptoms [1, 2]. To reduce mental health disparities in the US, it is important to evaluate whether depression varies by racial/ethnic groups. Progress has been made in the understanding of some racial and ethnic differences in depression for African Americans and Latinos. Latinos have been found to have higher prevalence rates of 12-month MDD compared to other ethnic groups [3]. African Americans have been found to have higher rates of persistence of MDD (lasting for 12 months within an individual’s lifetime), that the MDD is usually left untreated, that is more severe and more disabling compared to non-Hispanic Whites [4]. Furthermore, subgroup differences in depression among African Americans have been documented; Caribbean blacks have a higher rate of lifetime MDD prevalence compared to other African Americans [4].

Asian Americans (AA) constitute people with ethnic origins in the Far East, Southeast Asia, or the Indian subcontinent: Cambodia, China, India, Japan, Korea, Malaysia, Pakistan, the Philippine Islands, Thailand, and Vietnam [5]. AA is the fastest growing minority population in the US [5]. Paralleling the growing interest in global variations in the expression of clinical features of depression and non-Western treatments for depression, more studies are examining ethnic differences among Asians [6, 7]. Lee and colleagues reported ethnic variations in MDD onset among AA using nationally representative data and that poverty rate, age, and gender differently influenced the MDD onset among AA [8].

Although the prevalence of MDD among AA in community samples is reported to be moderate to low [9], high levels of depressive symptoms among AA adults have been described [10]. Among AA, depression tends to be very persistent, lasting long periods of time, and AA are less likely to seek treatment and adequate care compared to non-Hispanic Whites [11–13]. Depression may go under-recognized because of language and health literacy barriers, acculturation levels, or somatic presentations [14]. Yet, the overall prevalence of depression among various AA groups is still unclear. This is partially because some researchers often combine Asian with other minority groups in a category of ‘other’ or even exclude Asians from their studies [15, 16]. Thus, there is a need for a better understanding of depression among AA in the community in order to provide adequate mental and physical health services.

There are various methods to assess depression in community samples. Some tools enable depression diagnosis according to the definitions and criteria of ICD-10 and DSM-IV (e.g., Composite International Diagnostic Interview, CIDI), whereas other screening measures are based on general symptoms [e.g., Center for Epidemiologic Studies Depression Scale (CESD), Beck Depression Inventory (BDI), Hamilton Depression Inventory (HDI), Patient Health Questionnaire (PHQ), and Geriatric Depression Scale (GDS)]. A recent review of depression among AA attributed variation in prevalence to the instruments used to measure depression and by the context of each study [12]. AA who suffer from MDD may not report sadness or depressed mood as their primary complaint, and thus may be less likely to meet clinical criteria when using a tool such as the CIDI [12]. The most commonly used tool to assess depression among AA is the CESD. In this particular ethnic group, a high rate of somatic symptoms of depression—such as changes in appetite, headaches, backaches, stomachaches, insomnia, or fatigue—has been reported using the CESD [12].

The purpose of this study was to: systematically review the estimates of depression prevalence among various ethnic subgroups of AA in community samples, derive synthesized estimates of depression, and examine possible gender, ethnicity, other participant characteristics (e.g., age, parents for teenagers, maternity, homosexuals), and methodological factors associated with variation in these estimates. By providing an estimate of depression prevalence among AA in the community, the results of this study would be beneficial to the development of policies that aim to reduce mental health disparities in the US.

## Methods

The protocol and data extraction forms were designed in accordance with the Preferred Reporting Items for Systematic Reviews and Meta-Analysis: the PRISMA Statement [17, 18]. The following six databases were systematically searched using a comparable search strategy, with adapted terms for each database: PubMed, MEDLINE (OVID), CINAHL (EBSCOHost), PsychINFO (OVID), Web of Science (Web of Knowledge), and the Cochrane Library. Keywords were “Asian-Americans” and “depression/depressive symptoms”, and any matched subject headings or MeSH terms. For example, the search strategy in PubMed was: ((Asian Americans) OR Asian American)) AND (((((("Depression, Postpartum"[Mesh] OR "Depressive Disorder, Treatment-Resistant"[Mesh] OR "Depression, Chemical"[Mesh] OR "Depressive Disorder, Major"[Mesh] OR "Major Depressive Disorder 1" [Supplementary Concept] OR "Major Depressive Disorder 2" [Supplementary Concept] OR "Depression"[Mesh] OR "Depressive Disorder"[Mesh]))) OR depress*) OR depressive symptom) OR depression).

Inclusion criteria were as follows: full-text, published, peer-reviewed, English-language studies conducted in the US, target population of adults (≥ age 18), a reported prevalence level for depression, and conducted in a community setting. Studies published during the past 10 years (from 2004 to 2014) were included to provide a current prevalence of depression. Studies that did not provide a prevalence estimate or sufficient information from which a prevalence could be calculated, as well as those conducted in clinical care settings, were excluded.

Retrieved articles were exported to a reference manager and duplicates were hand-searched and removed. Two reviewers (H.J.K. and K.T.) independently reviewed all titles. After the first title review, the two reviewers independently reviewed selected abstracts. Full-text articles were selected when the two reviewers agreed that the article met the inclusion and exclusion criteria. H.J.K conducted the primary data extraction. All articles were examined independently by a second reviewer (K.T.). Inter-reviewer disagreement was minimal, and inconsistencies were discussed and resolved. References for all the articles were also scanned (citation tracking) for further relevant source papers, and similar procedures were used to include or exclude them.

Risk of bias in reporting depression prevalence in individual studies was assessed using the Agency for Healthcare Research & Quality (AHRQ) [19]. These criteria include sample size, methods for selecting participants, methods for measuring exposure variables, methods to deal with any design-specific issues such as recall or interviewer bias, and analytical methods to control for confounding factors. Because we focused only on sample characteristics and prevalence of depression for this review, four items: sample size (≥500 vs. <500), sampling methods (random vs. convienient), participation rate (reported vs. unreported or <50%), and eligibility criteria (provided vs. unprovided) were reviewed for each article. Total scores ranged from 0 to 4, with a smaller number indicating a higher risk of bias.

We performed meta-analyses to calculate pooled estimates of depression prevalence by method of measurement with Stata (version13.1, StataCorp LP, College Station, TX). Studies that used the standard questionnaire items and cut-points for each measure were included in the meta analysis. Heterogeneity—differences in the prevalence of depression across studies—arises when there are clinical or methodological differences between studies (i.e., participants characteristics, outcomes, or study design). This information is important in meta-analysis because the presence of heterogeneity can influence the pooled prevalence; high heterogeneity may produce misleading results [20]. Statistical heterogeneity was assessed using *I* ^2^, with thresholds of ≥ 25%, ≥ 50%, and ≥ 75% indicating low, moderate, and high heterogeneity, respectively [21]. To investigate heterogeneity, we performed subgroup analyses by gender (% of women), age, ethnicity, and other participant characteristics. Random-effects metaregression models (DerSimonian & Laird, with 95% CIs) were conducted to explore the subgroup differences with sample size as a covariate. We also conducted sensitivity analyses for prevalence of depression by including studies that used modified questionnaires or alternative cut-points, and by excluding studies with high risk of bias or with unreported participation rates. Egger’s tests of publication bias were performed to assess publication bias due to preferential publication of small studies reporting high prevalence estimates [22].

## Results

**Fig 1:**
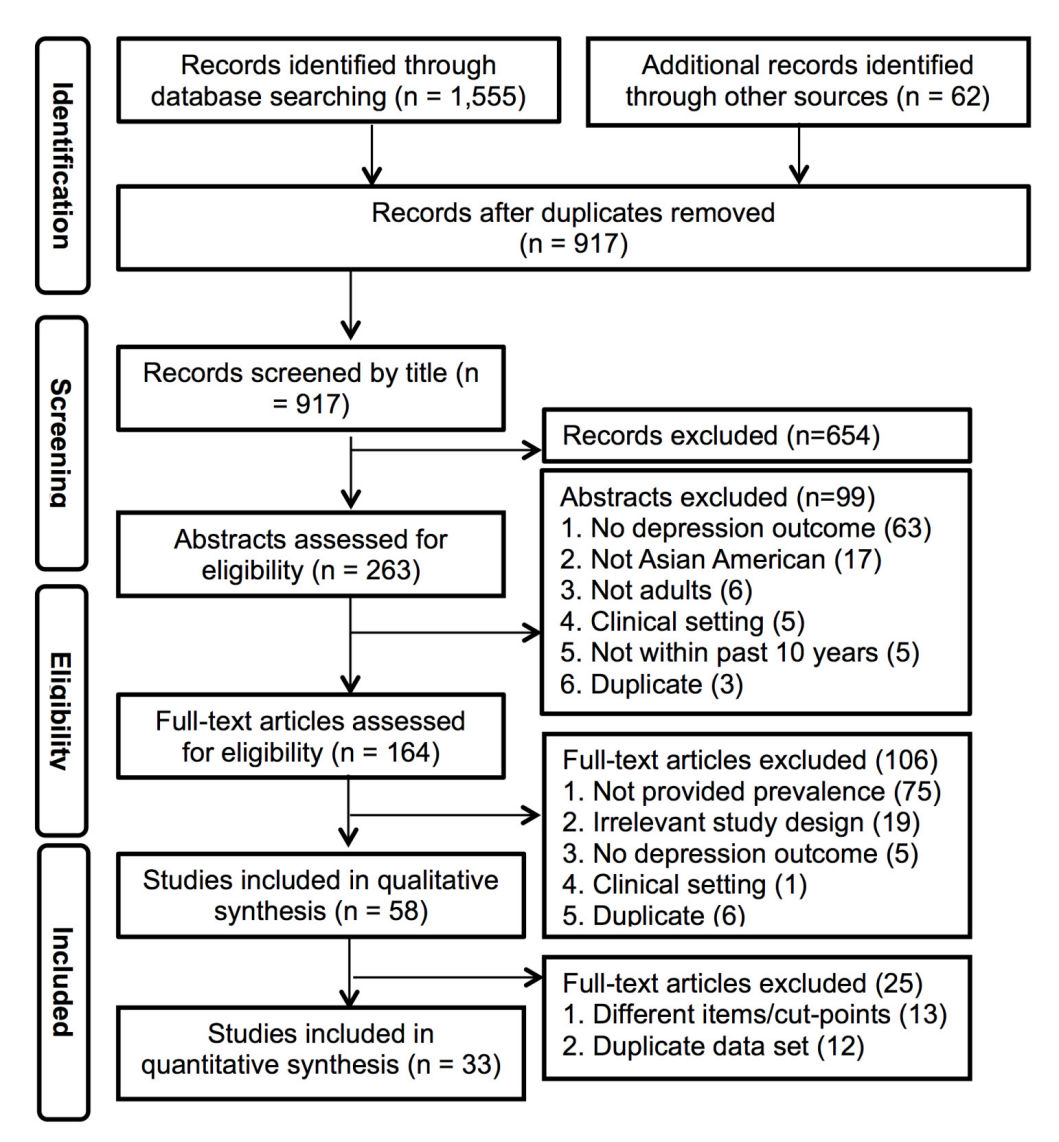
Flow diagram for review of studies of prevalence of depression in Asian Americans.

The literature search yielded 1,555 articles. An additional 62 articles were found through Google Scholar and relevant bibliographies. After removal of duplicates, the title screening process identified 263 potentially eligible studies (Fig 1). After abstracts were screened using the inclusion and exclusion criteria, 58 studies remained for qualitative synthesis (a total sample of 21,731 adults) [23–80].

The prevalence of depression and study characteristics of the selected articles are presented in Table 1. Multiple instruments have been used to assess depression. The most commonly used measure was the CESD, with 22 studies using this tool. The majority of the studies used the 20-item CESD and a score of 16 to classify depression. Jang and colleagues prefer a short form consisting of 10 items (with a cut-point of 10 demarcating depression), and these were included in the meta-analyses because a previous study indicated this is comparable to a score of 16 on the full version [81]. Three studies used modified versions of CESD, and one study used a different cut-point. Seven studies used the PHQ; one of the studies used only 2 items, while the others used 9 items in their assessment. Five studies used the GDS, 4 studies used the Hopkins Symptom Check List (HSCL), 2 studies used the BDI, and one study used the Postpartum Depression Screening Scale (PPDS). One study used screening stem questions of Fresno-CIDI, summed the symptoms that were under each criteria, and generated an estimate of lifetime depressive symptoms.

**Table 1:** Review of prevalence studies of depression in Asian-Americans.

Author	Year	Measurement	Cut point	Sample(N)	Sample (Education)	Sample (Ethnicity)	Sample (Characteristics)	Mean age (SD), Range	Women (%)	Study site	Risk of bias	Prevalence(%)
***Screening measures***												
Bromberger et al. [23]	2004	CESD-20	16	486	Chinese:73%; Japanese:83% (≥college)	Chinese, Japanese	Adults	42–52	100.0	Multiple site	3	14.2
Lam et al. [24]	2004	BDI	16 (cut off for college students)*	238	College students	Multiple (Chinese, Filipinos, Indian, Korean, and Vietnamese)	College students	18.8 (1.4)	59.5	New York, Binghamton	1	20.6
Lee & Farran [25]	2004	CESD-20	16	59	14yrs (SD = 3.3)	Korean	Caregivers	57.8 (12.2)	100.0	Chicago & LA area	1	71.0
Suen & Tusaie [26]	2004	GDS	14*	100	14yrs (SD = 5.66)	Taiwanese	Elderly	67.39 (6.99), 60–88	47.0	Northeastern city	1	7.0
Goyal et al. [27]	2005	PPDS-35*	80 (major), 60–79 (minor)	58	50% (≥Mater's degree), 47% (≥college)	Asian Indian	Maternity	29 (3.43), 22–38	100	No information	1	52; 24 (major), 28 (minor)
Jang et al. [28]	2005	CESD-10	10	230	58% (≥high school)	Korean	Elderly	69.8 (7.05), 60–92	59.1	Florida	1	30.0
Alderete et al. [29]	2006	Fresno-CIDI (screening stem questions)	Lifetime depressive symptoms	124	No Information	Multiple	Women with an abnormal mammogram	40–80	100.0	San Francisco Bay Area	1	34.7
Goebert et al. [30]	2006	CESD-20	16	39	74% (≥college)	Filipino, Japanese, Other	Maternity	27, 18–35	100.0	Hawaii	2	61.0
Mui & Kang [31]	2006	GDS	11	407	No Information	Multiple (Chinese, Korean, Indian, Filipino, Vietnamese, and Japanese)	Elderly	72.4 (6.2), 65–96	56.0	New York city	3	45.0
Suen & Morris [32]	2006	GDS	11	100	14yrs (SD = 5.66)	Taiwanese	Elderly	67.39 (6.99), older than 60	47.0	Northeastern City	1	16.0
Donnelly [33]	2007	PHQ-9	5	166	41% (≥college)	Korean	Adults	25–81	No information	East Coast	1	22.9
Tran et al. [34]	2007	CESD-19*	16	349	14yrs (SD = 6.17)	Vietnamese	Adults	38.76 (13.76), 18–73	52.1	East Coast metropolitan area	2	30.0
Birman & Tran [35]	2008	HSCL-25	1.75	212	10yrs (SD = 2.58)	Vietnamese	Adults, refugee	48.84 (7.14), 22–65	49.1	Washington DC, MD	1	20.8
Chae & Yoshikawa [36]	2008	CESD-20	16	192	No Information	Multiple (East Asian, South Asian, Southeast Asian, Filipino, and Other)	Gay	18–40s	0.0	Northeastern City	1	44.2
David [37]	2008	CESD-20	16	248	41% (≥college)	Filipino	Adults	28.4 (9.9), 18–66	48.8	Internet	1	29.8
Donnelly & Kim [38]	2008	PHQ-9	5	112	53% (≥college)	Korean	Elderly	57–81	No information	Northeast metropolitan area	1	36.0
Hwang & Goto [39]	2008	HDI-23	19	107	College students; years in college mean 2.92(SD 1.64)	Multiple (Chinese, Vietnamese, Japanese, Taiwanese, and Korean)	College students		66.4	Rocky Mountain region	1	14.0
Yoon & Lau [40]	2008	BDI-II	Clinical cut off*	140	College students	Multiple (South East, East-Chinese, Japanese, and Korean, and Filipino	College students	19.8 (2.05)	79.0	West Coast	1	17.0
Jang et al. [41]	2009	CESD-10	10	230	65% (≥high school)	Korean	Elderly	68.5 (6.40), 60–94	54.8	Florida	1	36.0
Kang et al. [42]	2009	GDS	11	120	No Information	Korean	Elderly	71.2 (5.0), 64–91	55.0	Arizona	1	38.1
Kim [43]	2009	CESD-20	16	78	15 yrs (SD = 2.58)	Korean	Adults	43.68 (4.25), 34–57	62.8	Pacific Northwest	1	30.0
Cheung et al. [44]	2010	HSCL-25	1.75	205	68.3% (≥college)	Korean	Adults	44 (11.0)	55.0	Huston, Texas	1	18.5
Hwang et al. [45]	2010	HDI-23	19	105	No Information	Chinese	Parents for teenagers	47.32 (4.67), 36–60	100.0	Western US	1	4.5
Kim et al. [46]	2010	CESD-20	16	172	15 yrs (SD = 3.04)	Korean	Parents for teenagers	40.90 (3.53)	69.2	Pacific Northwest	1	30.1
Li & Hicks [47]	2010	CESD-20	16	168	65% (≥college)	Chinese	Adults	34 (12.0)	100.0	Boston	3	26.0
Bernstein et al. [48]	2011	CESD-20	21*	304	65%(≥college)	Korean	Adults	46.7 (14.3)	56.6	New York	1	13.2
Hahm et al. [49]	2011	CESD-20	16	400	86% (≥college)	Korean, Chinese, Vietnamese, Other	Adults	18–35	100.0	Massachusetts	2	31.0
Herman et al. [50]	2011	CESD-20	16	589	College students	Japanese, Filipino, Other	College students	19.7 (4.0), 18–53	67.2	Hawaii	3	38.5
Jang et al. [51]	2011	CESD-10	10	675	70% (≥high school)	Korean	Elderly	70.2 (6.87), 60–96	58.8	Florida	2	30.8
Kim [52]	2011	CESD-20	16	99	Female:15yrs(SD = 2.78); males:16yrs(SD = 3.17)	Korean	Parents for teenagers	F: 42.20 (3.68), M: 45.06 (4.21)	64.6	Pacific Northwest	1	28.4
Berg et al. [53]	2012	PHQ-2*	3	495	70% (≥college)	Multiple	Adults	32	48.6	Minnesota	3	4.9 (Smoker 19.6)
Harada et al. [54]	2012	CESD-11	9*	3139	No Information	Japanese	Elderly	71–93	0.0	Multiple site	2	9.7
Huang et al. [55]	2012	CESD-12 or CIDI-SF	No information*	1150	70% (≥college)	Multiple	Maternity	US born 29.2 (.71); Foreign-born 31.2 (0.26)	100.0	Multiple site	4	4.57 (US-born 6.8; Foreign-born 4.1)
Kim [56]	2012	CESD-20	16	72	Females:15yrs(SD = 3.52), males:16yrs(SD = 1.66)	Korean	Parents for teenagers	F: 37.16 (3.88), M: 39.58 (5.53)	73.6	Pacific Northwest	1	29.6
Leung et al. [57]	2012	HSCL-25	1.75	516	77% (≥college)	Chinese	Adults	48.3 (18.1)	56.8	Huston, Texas	1	17.4
Park & Rubin [58]	2012	CESD-20	16	516	83% (≥college)	Korean	Adults	39.36 (9.32), 21–82	51.6	California and Texas	2	48.0
Cheung et al. [59]	2013	HSCL-25	1.75	43	65% (≥college)	Japanese	Adults	38.3 (12.2)	56.0	Huston, Texas	1	11.6
Lemieux et al. [60]	2013	CESD-20	16–26 (mild depression), 27+ (major depression)	319	33% (>college)	Multiple (Chinese, Filipino, Vietnamese, and Other Asians)	Men who have sex with men	31.3 (7.8)	0	Washington, DC & Philadelphia, PA	1	mild: 49.8, major depression: 11.60
Park et al. [61]	2013	PHQ-9	5	363	67% (≥college)	Korean	Adults	46.33 (14.16), 18–81	57.85	New York City	1	23.1% (mild)
Camacho et al. [62]	2014	CESD-20	16	784	No Information	Chinese	Adults	45–84	No information	Multiple site	2	8.16
Chen et al. [63]	2014	PHQ-9	5–9 (mild), 10–14 (moderate), 15–19 (moderately severe), and 20–27 (severe)	113	Undergraduate/graduate students	Multiple (Asian American or Pacific Islander)	Students in degree program (undergraduate vs graduate program)	24.99 (4.24), 18–35	42.5	No information	2	22.8 (mild), 10 (moderate), 2.7 (moderately severe or severe)
Dong et al. [64]	2014	GDS-5*	No information*	78	10.7 yrs	Chinese	Elderly	74.8 (7.8)	52	Chicago	1	21.8
Dong et al. [65]	2014	PHQ-9	1–4 (minimal), 5–9 (mild), 10–14 (moderate), 15–19 (moderately severe), and 20–27 (severe)	3159	30% (9–12 yrs), 21% (≥13yrs)	Chinese	Elderly	60 years and above	58.9	Multiple site	4	37.3 (minimal), 13.3 (mild), 2.8 (moderate), 1.1 (severe)
Lee et al. [66]	2014	PHQ-9	5 (any depression), 10 (moderate to severe)	630	10.9 (4.4)	Korean	Elderly	70.9 (6.1)	68.9	Baltimore—Washington metropolitan area	3	23.2 (any depression), 7.3 (moderate to severe)
***Standardized clinical interview***												
Hwang & Myers [67]	2007	UM-CIDI	Lifetime MDE*	1747	13yrs (SD = 3.78)	Chinese	Adults	38.38 (12.65), 18–65	49.6	Los Angeles County	4	6.9
Gavin et al. [68]	2010	WMH-CIDI	Lifetime & 12 month MDE	2178	Female:38% (≥16yrs), male: 46% (≥16yrs)	Multiple (Chinese, Filipino, Vietnamese, and Other Asian)	Adults	M: 40.85(0.90), F: 42(0.76)	52.5	CPES (NLAAS) data**	4	4.7
Jimenez et al. [69]	2010	WMH-CIDI	12 month MDD (DSM-IV)	260	30.4% (≤11yrs), 19.2% (12yrs), 17.7% (13–15yrs), 32.7% (≥16yrs)	Multiple	Elderly	60 years and above	No information	CPES (NLAAS) data**	3	Lifetime MDE 7.5, lifetime any depressive disorder 7.7, 12 month MDE 2.1, any depressive disorder 2.1
Chou et al. [70]	2011	WMH-CIDI	Lifetime MDD	793	No Information	Multiple	Adults who reported racial discrimination experiences	39.4 (13.5)	47.0	NLAAS data**	3	8.8
Chae et al. [71]	2012	WMH-CIDI	12 month MDD	2095	Female:38% (≥16yrs), male: 46% (≥16yrs)	Multiple (Chinese, Filipino, Vietnamese, and Other Asian)	Adults	Male: 40.85(0.90), Female: 42(0.76)	52.5	NLAAS data**	4	4.7
John et al. [72]	2012	WMH-CIDI	12 month MDD (DSM-IV)	1530	20.2% (≥17yrs), 49.9% (13–16 yrs)	Multiple (Chinese, Filipino, Vietnamese, and Other Asian)	Adults, being currently employed or unemployed but looking for work	39	47.64	NLAAS data**	4	5
Sangalang & Gee [73]	2012	WMH-CIDI	12 month MDD (DSM-IV-TR)	2066	Some college (25.07%), college graduate (42.87%)	Multiple (Chinese, Filipino, Vietnamese, and Other Asian)	Adults	41.27 (0.70), 18–95	52.59	NLAAS data**	4	4.6
Lee et al. [74]	2013	WMH-CIDI	Lifetime MDD	1280	59.5% (>High school)	Multiple (Vietnamese, Filipino, and Chinese)	Adults	44.8 (0.5)	55.4	CPES (NLAAS) data**	3	6.8
Zhang et al. [75]	2013	WMH-CIDI	Lifetime and 12 month MDD	600	50% (≥16yrs)	Chinese	Adults	41.59 (0.57), 18–85	52.7	NLAAS data**	3	Lifetime 11.3%, 12 month 7.5%
Alegria et al. [76]	2014	WMH-CIDI	12 month psychiatric disorder, any depressive (dysthymia, MDD), DSM-IV	2095	42% (≥16yrs)	Multiple	Adults	18–34 (39.5%), 35–49 (32.2), 50–64 (18%), 65 and over (10.3%)	52.5	CPES (NLAAS) data**	4	5.01
Kalibatseva et al. [77]	2014	WMH-CIDI	Lifetime MDE (DSM-IV)	310	49.2% (≥16yrs)	Multiple (Vietnamese, Filipino, Chinese, and Other Asian)	Adults, screened as endorsed depressed mood	39.22 (0.88)	61.3	CPES (NLAAS) data**	2	69
Kim & Lopez [78]	2014	WMH-CIDI	Lifetime and 12 month MDD (ICD-10 & DSM-IV)	310 (screened), 2095 (total)	No Information	Multiple (Chinese, Filipino, Vietnamese, and Other Asian); screened as endorsed depressed mood	Adults	38.7 (14.1)-screened, 41.0 (14.7)-total	61 (screened), 53 (total)	NLAAS data**	4	Screened- depressed 89%; discouraged about life 84.2%; lost interest in enjoyable things 73.9%; Total- lifetime MDD: 9.2, 12 month MDD: 4.5
Park et al. [79]	2014	WMH-CIDI	12 month MDE (DSM-IV)	164	Some college (15.68%), college graduate (30.46%)	Multiple	Elderly age 65+	72.35	57.49	NLAAS data**	3	2.59
Tan [80]	2014	WMH-CIDI	Lifetime MDD & MDE, 12month MDD & MDE	487	No Information	Chinese (those who immigrated as children were excluded)	Adults	No information	No information	CPES (NLAAS) data**	2	US born, immigrants, respectively; Lifetime MDD: 20.7, 7.2; 12month MDD: 8.1, 3.7; lifetime MDE: 21.1, 7.8; 12month MDE: 8.6, 3.7

* Excluded for meta-analysis due to different items/cut-points;

** Excluded for meta-analysis due to duplicate database; BDI: Beck Depression Inventory; CESD: Center for Epidemiologic Studies of Depression; HDI: Hamilton Depression Inventory; PDSS: Postpartum Depression Screening Scale; WMH-CIDI: World Mental Health Organization Composite International Diagnostic Interview; UM-CIDI: University of Michigan’s version of CIDI; PHQ: Patient Health Questionnaire; GDS: Geriatric Depression Scale; HSCL: Hopkins Symptom Checklist; MDD: Major Depressive Disorder; MDE: Major Depressive Episode; NLAAS: National Latino and Asian American Study; CPES: Collaborative Psychiatric Epidemiology Surveys, the CPES includes the National Comorbidity Survey-Replication, the NLAAS and the National Survey of American Life.

In total, 14 studies were found to use a depression measure based on standardized clinical criteria (Table 1); however, these studies were not included in the meta-analyses because 13 of the studies used the same database, the National Latino and Asian American Study (NLAAS). The other study by Hwang & Myers [67] reported lifetime Major Depressive Episode (MDE) with DSM-IV criteria which was different from other studies; therefore, pooled prevalence of depression measured by standardized clinical approaches were not estimated.

In regard to the risk of bias of individual studies, nearly half of the studies, 28 out of 58, had a score of 1, which indicates a high risk of bias. Specifically, the sample size for 39 studies was less than 500; 38 studies used non-random/convinient sampling methods. Several of the studies sampled from a restricted geographical area, while others represented multiple sites (Table 1). However, both the east and west coasts of the US were represented. Overall, the sample sizes ranged from 39 to 3,159 participants [median = 254; interquartile range (IQR) 120–630]. Most studies included both female and male participants in their samples; 9 studies included only female participants, and one study included only males. The median percentage of females represented in the sample was 56% (IQR 52.0–67.2).

The median of mean ages was 41 years [IQR 38.0–48.9]. Several studies focused on the elderly, 5 studies looked at college students’ depressive symptoms, while others were maternal and parental studies or studies of caregivers. Two studies looked at homosexual AA’s depressive symptoms. While 26 studies included AA from multiple ethnicities, the samples in 16 studies were limited to Koreans, and 8 studies were limited to Chinese. Two studies each for Japanese, Taiwanese, and Vietnamese, and 1 study each for Filipino, and Indian were found.

### Prevalence of depression

**Fig 2:**
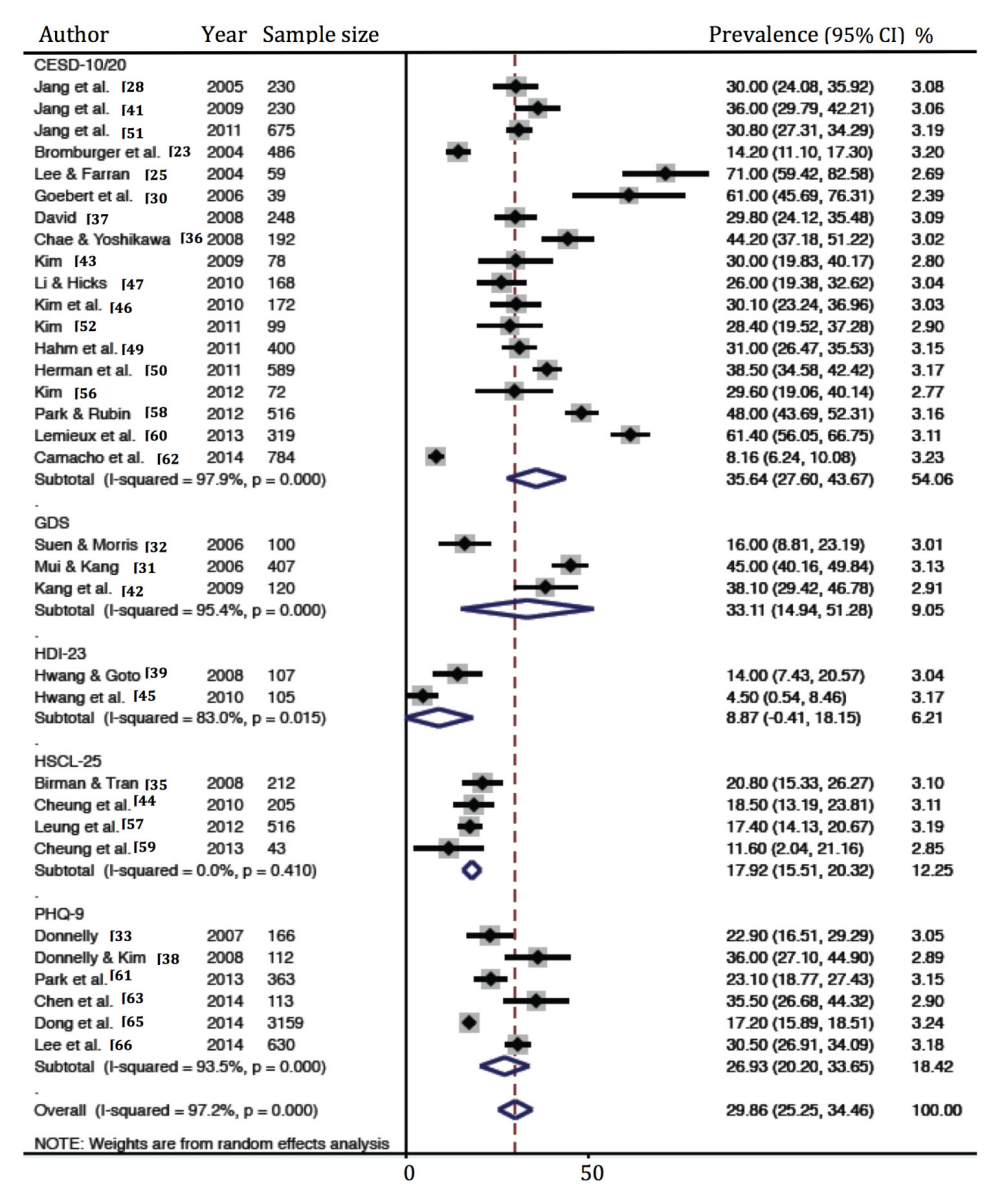
Prevalence of depression in Asian Americans. CESD, Center for Epidemiologic Studies Depression Scale; GDS, Geriatric Depression Scale; HDI, Hamilton Depression Inventory; HSCL, Hopkins Symptom Check List; PHQ, Patient Health Questionnaire.

The prevalence rate of depression ranged between 2.6% and 71.0% in individual studies (Table 1). The summary of the prevalence rate of depression by the various tools used to assess it is presented in Table 2. The meta-analytic pooled prevalence rate of depression (Fig 2) using the CESD was estimated to be 35.6% (95% CI 27.6%, 43.7%), with high heterogeneity (*I* ^2^ = 97.9%). The prevalence rate of depression measured by the GDS, with a threshold of 11, was 33.1% (95% CI 14.9%, 51.3%), with high heterogeneity (*I* ^2^ = 95.4%). The prevalence rate of depression measured with the PHQ-9, with a threshold of 5 indicating mild depression, was 26.9% (95% CI 20.2%, 33.7%), with high heterogeneity (*I* ^2^ = 93.5%). A relatively low prevalence rate of depression, 8.9% (95% CI-.4%, 18.2%), was estimated via HDI with a threshold of 19, which is a major depressive clinical cutoff, with high heterogeneity (*I* ^2^ = 83%). A prevalence of 17.9% (95% CI 15.5%, 20.3%) with low heterogeneity (*I* ^2^ = 0%) was measured by HSCL. Significant publication bias, according to the Egger’s test, was found in the analysis [Egger’s bias = 6.36 (95% CI 5.24%, 10.10%), P < 0.001].

**Table 2:** Summary of depression prevalence by measurement type.

Measurement	Definition/cut-point	No. of studies	No. of participants	Prevalence, % (95% CI)	Hetero-geneity, I^2^ (%)
***Standardized clinical interview***					
CIDI					
UM-CIDI	Lifetime MDE	1	1747	6.9	
WMH-CIDI	Lifetime & 12 month MDD/MDE	13*	2095**	Lifetime MDD: 9.2,12 month MDD: 4.5**	
*Screening measures*					
Fresno-CIDI (screening questions)	Lifetime depressive symptoms	1	124	34.7	
HDI-23	19 (Major depression clinical cutoff)	2	212	8.9 (-.4, 18.2)	83.0
PHQ					
PHQ-2	3	1	495	4.9	
PHQ-9	5	6	4543	26.9 (20.2, 33.7)	93.5
PPDS	60	1	58	52.0	
HSCL-25	1.75	4	976	17.9 (15.5, 20.3)	0.0
GDS					
GDS-30	14	1	100	7.0	
GDS-30	11	3	627	33.1 (14.9, 51.3)	95.4
GDS-5	Not presented	1	78	21.8	
BDI					
BDI	16	1	238	20.6	
BDI-II	Clinical cut off	1	140	17.0	
CESD					
CESD-10/20	10/16	18	5356	35.6 (27.6, 43.7)	97.9
CESD-11	9	1	3139	9.7	
CESD-19	16	1	349	30.0	
CESD-20	21	1	304	13.2	

*Meta-analysis was not conducted due to duplicate dataset;

**data from Kim & Lopez [78]; UM-CIDI: University of Michigan’s version of Composite International Diagnostic Interview; WHM-CIDI: World Mental Health Organization CIDI; HDI: Hamilton Depression Inventory; PHQ: Patient Health Questionnaire; PDSS: Postpartum Depression Screening Scale; HSCL: Hopkins Symptom Checklist; GDS: Geriatric Depression Scale; BDI: Beck Depression Inventory; CESD: Center for Epidemiologic Studies of Depression.

### Sensitivity and subgroup analysis

The results of sensitivity and subgroup analyses of the pooled prevalence rates of depression by measurement are shown in Table 3. There was no significant pattern in the sensitivity analyses. The prevalence rates of depression did not differ statistically by the percentage of women or between adults and elderly; subgroup effects based on gender and age did not explain the high statistical heterogeneity found in the primary analysis; however, the pooled prevalence rate of depression measured by CESD of special populations, which included maternity, caregivers, and homosexuals, was 58.8% (95% CI 47.2%, 70.4%). This prevalence rate was significantly higher than that of the estimate based on other groups (*p* = .003).

**Table 3:** Subgroup and sensitivity analyses.

	CESD		GDS		PHQ	
**Primary analysis**	35.6 (27.6, 43.7), I^2^ = 97.9%, 18 studies (n = 5356)		33.1 (14.9, 51.3), I^2^ = 95.4%, 3 studies (n = 627)		26.9 (20.2, 33.7), I^2^ = 93.5%, 6 studies (n = 4543)	
**Sensitivity analyses**						
Including studies that used modified or different items/cut-points	32.9 (26.2, 39.6), I^2^ = 98.4%, 21 studies (n = 9148)		25.6 (8.9, 42.2), I^2^ = 96.9%, 5 studies (n = 805)		23.7 (16.0, 31.5), I^2^ = 97.6%, 7 studies (n = 5038)	
Excluding studies at high risk of bias	30.6 (17.6, 43.6), I^2^ = 67.2%, 8 studies (n = 3657)				27.2 (15.8, 38.7), I^2^ = 59.9%, 3 studies (n = 3902)	
Excluding studies with unreported participation rate or <50%	35.2 (21.3, 49.1), I^2^ = 58.3%, 6 studies (n = 2198)				24.1 (16.1, 32.1) I^2^ = 90.8%, 4 studies (n = 3636)	
**Subgroup analyses**						
		Sig*		Sig*		Sig*
% of women		p = .736		p = .072		p = .838
Age group						
Adults	36.4 (26.7, 46.1), I^2^ = 98.2%, 15 studies (n = 4221)	p = .876			26.3 (19.7, 32.9), I^2^ = 69.6%, 3 studies (n = 642)	p = .179
Elderly	31.8 (28.7, 34.8), I^2^ = 17.2%, 3 studies (n = 1135)				27.4 (15.9, 38.9), I^2^ = 96.7%, 3 studies (n = 3901)	
Other participant characteristics						
Parents for teenagers	29.5 (24.7, 34.3), I^2^ = .0%, 3 studies (n = 343)	p = .659				
Special population	58.8 (47.2, 70.4) I^2^ = 85.9%, 4 studies (n = 609)	p = .003				
Others	29.3 (20.4, 38.1), I^2^ = 98.0%, 11 studies (n = 4404)	reference group				
**Subgroup analyses**						
Ethnicity (Excluded special population)						
Korean	33.3 (27.5, 39.1), I^2^ = 85.8%, 8 studies (n = 2072)	p = .012	31.0 (17.2, 44.8), I^2^ = 80.9%, 2 studies (n = 200)		27.6 (22.2, 33.0), I^2^ = 75.3%, 4 studies (n = 1271)	
Chinese	15.7 (6.5, 24.9), I^2^ = 93.2%, 3 studies (n = 1176)	Reference group				
Japanese	20.4 (6.9, 34.0), I^2^ = 86.4%, 2 studies (n = 355)	p = .646				
Filipino	34.4 (23.2, 45.6), I^2^ = 66.6%, 2 studies (n = 313)	p = .049				

*Random-effects meta-regression models (with 95% CIs) were conducted to explore the subgroup differences (DerSimonian and Laird) with sample size as a covariate; CESD: Center for Epidemiologic Studies of Depression; GDS: Geriatric Depression Scale; PHQ: Patient Health Questionnaire.

Group differences among different ethnic groups were identified. The pooled prevalence rate of depression measured by CESD, excluding the special populations—maternity, homosexual, and caregivers—for Koreans was 33.3% (95% CI 27.5%, 39.1%) and this was significantly higher than that for Chinese (15.7%, 95% CI 6.5%, 24.9%, *p* = .012). For Filipinos, the prevalence rate was estimated to be 34.4% (95% CI 23.2%, 45.6%), and this was also significantly higher than that for Chinese (*p* = .049). The prevalence rate of depression was estimated to be 20.4% (95% CI 6.9%, 34.0%) for Japanese, which was not significantly different than that for Chinese.

## Discussion

This is the first study to estimate the prevalence rate of depression among AA adults in the community using synthesized data obtained from a systematic review of the literature published in the past 10 years. There was variation in the pooled estimates of prevalence by the type of measure used to assess depression, but we found that depression is highly prevalent in AA. The pooled estimates from this review indicate that 35.6% of AA have depressive symptoms as assessed by the CESD, 33.1% by the GDS and 26.9% by the PHQ. These estimates are comparable to those found among patients with chronic disease [82]. Studies indicate that 4.5% to 11.3% of adult AA in the community meet the criteria for major depression, but the majority of estimates come from the same dataset. The differences of magnitude between estimates obtained from symptom screening versus standardized clinical approaches were expected, and this result is consistent with a similar review of depression in a different population [82]. However, attention should be given to the wide gap between the two estimates. As stated above, it has been reported that AA are less likely to be diagnosed with a mental disorder [12]. Further, it has been documented that AA with depression are less likely to access any depression treatment or to receive adequate mental health care compared to non-Hispanic Whites [10, 12]. This is also related to lower rates of detection and treatment of depression, which may lead to a worse prognosis. Given that depressive symptoms are highly prevalent among AA in the US, greater effort should be given to establishing public health policy programs that increase access to mental health care, including adequate screening and a referral system. We also suggest studies linking this issue to health insurance coverage.

We found no gender effect on the prevalence rate of depression from this review. Inconsistent findings have been reported in previous AA depression studies. While no male-female differences were seen in a study by Yeung et al. [83], depressive symptoms have been found to be higher among Asian women than men [84]. Our review also did not find any age group differences. These results may be influenced by the complex and diverse sample characteristics of each study in this review. Specifically, subgroup analysis revealed that the prevalence rate of depression of special population studies that included maternity, homosexual, and caregivers were significantly higher than other groups. Similar findings have been found in non-AA populations such that lesbian, gay, and bisexual groups have higher rates of depression [85]. Depression of perinatal women has been studied and the importance of depression screening for this group has been addressed previously [86]. A recent prospective study found that spousal caregivers of persons with dementia have a high risk of developing a depressive disorder [87]. These findings suggest that some populations could be prioritized in public mental health interventions to prevent and screen for the occurrence of depression. This may also apply to underserved ethnic minorities, including AA, as significant health disparities persist in diverse communities across the US.

Another factor that explains the heterogeneity of the prevalence rate of depression would be ethnicity. The prevalence rate of depression measured by CESD from the studies excluding the special population—maternity, homosexual, and caregivers—was higher among Korean and Filipino subgroups, but not in Japanese, compared to Chinese. The emotional characteristics of Koreans—the feeling of regret regarding neglect of children or parents that would be labeled *guilt* or *shame*—has been reported to be associated with depressive symptoms in Koreans [88]. Interestingly, according to a cross-national comparison study of depressive symptoms between Japanese and Whites, Japanese respondents tend to have lower mean scores on the CESD than Whites [89]. The prevalence rate of diagnostic major depression among Chinese-Americans has been found to be higher [75] than the average among the total sample of the NLAAS [78]. Our findings in this review suggest that differences in the prevalence rates of depression exist among Asian ethnicity groups. Heterogeneity of AA ethnic groups in sociohistorical, cultural, economic, and political characteristics has previously been reported [12]. Further studies providing information about the subgroups of AA in this regard are required to build evidence to develop strategies for preventing or reducing depression in AA.

## Limitations

The high estimated prevalence rate of depression in AA from this review should be interpreted with caution. Heterogeneity across the studies was considerably high, and the samples were very diverse in age and context. In addition, information related to depression in this population, such as immigration status, length of stay in the US, English language proficiency, and other psychological status (e.g., acculturation, racial discrimination) [12, 14, 90], was not synthesized in this review because of lack of information or heterogeneity of such information across the studies. Significant publication bias was found using the Egger’s test. Small studies with low prevalence may be less likely to be published than studies reporting a high prevalence of depression. Generally, the studies in this review had a high risk of bias in reporting prevalence of depression. Most studies included in the meta-analysis used non-random sampling methods; thus, it is difficult to generalize the results to the total population of AA in the US. However, the problem of sampling AA due to the geographical distribution of Asians in the US has been noted. Outside of the major states of California and New York, obtaining satisfactory samples of AA using random sampling techniques is challenging [12]. This study fills a gap in the literature by providing an estimated aggregated prevalence rate of depression in AA using studies that selected samples from non-clinical settings from relatively diverse areas in the US. Non-standardization of methods, such as the measure used to assess depression across studies also detracts from the findings. For example, the CESD (developed in 1977), the most commonly used tool, does not cover current diagnostic DSM criteria of depression, and contains items such as the perceptions of others, talkativeness, or comparisons with others that are not necessarily related to depression [91].

## Conclusion

We have systematically reviewed estimates of the prevalence rate of depression among noninstitutionalized AA and found a wide range of heterogeneity in depression estimates among AA adults. Specific subgroups of the population, such as homosexual, caregiver, and perinatal woman, were more likely to be depressed. Possible variations by ethnicity were also noticed. Practitioners and researchers who serve AA adults need to be sensitive to the potential diversity of depression expression and treatment-seeking across AA subgroups, and pay further attention to better recognize those suffering from depression. Studies examining health insurance coverage and access to medical care for AA are required to provide the evidence needed to establish more effective public health or public policy programs to better recognize of depression, and to increase access to mental health care for AA. Such studies and policy programs may ultimately lead to a decrease in the variation in the prevalence of depression among ethnic minorities and curb treatment disparities.

## Supporting Information

S1 File 2.1.1—PRISMA 2009 Checklist. (DOC)
